# A semi exact solution for a metallic phase in a Holstein-Hubbard chain at half filling with Gaussian anharmonic phonons

**DOI:** 10.1038/s41598-021-91604-6

**Published:** 2021-06-10

**Authors:** Debika Debnath, M. Zahid Malik, Ashok Chatterjee

**Affiliations:** School of Physics, University of Hyderabad, Hyderabad, 500046 India

**Keywords:** Materials science, Physics

## Abstract

The nature of phase transition from an antiferromagnetic SDW polaronic Mott insulator to the paramagnetic bipolaronic CDW Peierls insulator is studied for the half-filled Holstein-Hubbard model in one dimension in the presence of Gaussian phonon anharmonicity. A number of unitary transformations performed in succession on the Hamiltonian followed by a general many-phonon averaging leads to an effective electronic Hamiltonian which is then treated exactly by using the Bethe-Ansatz technique of Lieb and Wu to determine the energy of the ground state of the system. Next using the Mott–Hubbard metallicity condition, local spin-moment calculation, and the concept of quantum entanglement entropy and double occupancy, it is shown that in a plane spanned by the electron–phonon coupling coefficient and onsite Coulomb correlation energy, there exists a window in which the SDW and CDW phases are separated by an intermediate phase that is metallic.

## Introduction

The exotic superconducting behaviour in ceramic Cu-based compounds^1^ has continued to elude a convincing theoretical paradigm that could consistently conform to all experimental observations. Though a large number of investigators have posed their faith in the electronic mechanism, there have also been quite a few advocates of the phonon mechanism^2–7^ and this tribe has grown with time. The main objection against the phonon mechanism is as follows. In a strongly correlated system, if the electron–phonon (e-p) coupling is small, the minimum energy state will have the characteristics of a polaronic spin-density-wave (SDW) state that corresponds to an antiferromagnetic (AFM) Mott insulator. Naturally for the superconductivity to be driven by the phonon-mechanism, the e-p coupling needs to be adequately large compared to the electron–electron (e-e) repulsive Coulomb correlation strength. A study by Plakida^8^ has shown that the lattice instability and strong e-p interaction have a pivotal role in inducing high ${T}_{C}$ superconductivity. Interestingly, however, if the e-p coupling is strong, the ground state (GS) of the system is described by the bipolaronic charge-density-wave (CDW) which corresponds to a paramagnetic Peierls insulator^9,10^.

The Holstein-Hubbard (HH) model is a suitable model to study the interplay between the e-e interaction and e-p interaction. A Monte-Carlo study by Fradkin and Hirsch^9^ on this model has revealed that with increasing strength of the e-p coupling, the GS of the HH model makes a direct SDW-CDW transition. The prospect of the phonon mechanism as the cause of inducing pairing in high-temperature superconductors appears to have been impeded to a great extent by such observations.

Interestingly, the theoretical observation of Takada and Chatterjee (TC)^10^ in 2003 has thrown a new challenge in the field. TC have looked into the SDW-CDW transition in the one-dimensional (1D) half-filled HH model analytically in a more careful way. They have treated the phonon subsystem variationally and the effective electronic Hamiltonian exactly by the Bethe ansatz method of Lieb and Wu (LW)^11^. The principal premise of the investigation of TC is as follows. With the increase in e-p coupling, both the effective onsite e-e interaction energy $({U}_{eff})$ and the effective hopping energy $({t}_{eff})$ decrease and with ${U}_{eff}$ approaching zero, the system becomes so sensitive to the interplay between the relative strengths of these two energy scales that instead of going from a SDW phase to a CDW phase, the system prefers to settle in an intermediate phase which has been shown by TC to be metallic. This intriguing observation of TC has sparked off a lot of interest in this issue and naturally, a host of investigations^12–15^ followed closely on the heels of the work of TC. Using the density matrix renormalization group (DMRG) technique, Clay and Hardikar^12,13^ have not only demonstrated the existence of an intermediate metallic phase in concordance with the contention of TC but also suggested that this intermediate phase can exhibit superconductivity, which is an exciting result in the context of high-temperature superconductivity. Feshke et al.^14^ have also implemented the DMRG method and established the occurrence of the metallic regime between the two insulating phases. They have also proved that intermediate metallic regime widens as the phonon frequency increases. Several other studies using the renormalization group (RG) technique^16^, Monte-Carlo simulations^17^, exact numerical diagonalization and cluster perturbation theory^18^ etc. have also shown the evidence of the intervening metallic phase between the SDW and CDW states.

Tezuku et al.^19^ have studied the HH model with strong e-e and strong e-p interactions with DMRG. They have considered the region between the anti-adiabatic and adiabatic limits and observed that when the e-e and e-p interactions are of comparable strength, the pairing and CDW correlations are degenerate. They have furthermore shown that when the phonon energy scale is much higher than the e-p interaction scale and the electron–hole symmetry is broken, the on-site superconducting phase overlaps with CDW state and then the transition from SDW to CDW does not require any intermediate phase. In a modified study, Tezuka et al.^20^ have calculated the correlation functions with the real space dynamics. Though for the pure (un-doped) HH model, they have found a metallic region in between the SDW-CDW phases, for the doped HH model with broken electron–hole symmetry, they have found the pairing correlation to be more dominant.

Tam et al.^21^ have studied the 1D HH model at half-filling using renormalization group (RG) method. They have considered the e-p interaction and e-e interaction on the same footing and considered all the possible retardation effects of the phonon dynamics. Their study using mean-field RG suggests a direct transition from the CDW state to the SDW state.

The above studies suggest that more detailed analytical investigations are required to predict unequivocally the existence of the intermediate metallic phase at the CDW-SDW cross-over region. To that end, Chatterjee and collaborators^22–28^ have carried out a few improved variational calculations and have shown that with every improvement of the variational wave function, the width of the intermediate metallic phase increases. This certainly lends a fair amount of credence to the original prediction of TC.

Chatterjee and Takada (CT)^28^ have also examined the problem in the presence of lattice anharmonicity. They have considered cubic and quartic phonon anharmonicities and have shown that the metallic phase becomes broader in the presence of lattice anharmonicity and thus the conjecture on the presence of the intervening metallic regime in the HH system is strengthened in the presence of anharmonic phonons. This work is of much importance because lattice anharmonicity has been found to play a crucial role in high ${T}_{c}$ superconductors. In fact, it has been observed that apex oxygen has a substantial anharmonic motion in the cuprates and also the phonon anharmonicity makes a significant impact on the electronic structure of these systems^29–33^. Konior^34^ has explained the importance of Gaussian phonon anharmonicity in the context of high ${T}_{c}$ superconductors and have shown that in the presence of this anharmonicity, the hopping parameter reduces at a slow rate causing an enhancement in the polaron mobility and the polaron bandwidth, which is a favourable condition for the phonon mechanism to stake a claim for inducing pairing. Lavanya, Sankar and Chatterjee (LSC)^25^ have recently re-examined the work of CT with Gaussian anharmonic potential by applying in succession a number of unitary transformations followed by an averaging with a general many-phonon state and the Bethe ansatz technique. This gives a wider metallic phase.

The principal aim of the present paper is to further modify the variational wave function of the phonon sub-system used by LSC for the anharmonic HH system to obtain a better solution for the GS energy and the SDW-CDW phase diagram. This calculation can be considered as semi-exact as we have included rigorously all possible phonon processes including coherence and correlations while treating the phonon subsystem and solved the electronic part exactly with the help of the Bethe ansatz technique and LW’s solution. The GS energy, local spin moment, von Neumann entropy, the double occupancy parameter and the phase diagram at the SDW-CDW transition region have been obtained.

## The Model

A one dimensional HH system with Gaussian phonon anharmonicity may be described by the Hamiltonian

1 $$H = H_{e} + H_{p} + H_{ep} ,$$

with

2 $$H_{e} = - t\mathop \sum \limits_{{\left\langle {ij} \right\rangle \sigma }} c_{i\sigma }^{\dag } c_{j\sigma } + U\mathop \sum \limits_{i} n_{i \uparrow } n_{i \downarrow } ,$$

3 $$H_{p} = \hbar \omega _{0} \mathop \sum \limits_{i} b_{i}^{\dag } b_{i} + \lambda _{{ap}} \mathop \sum \limits_{i} e^{{ - \gamma \left( {b_{i}^{\dag } + b_{i} } \right)^{2} }} ,$$

4 $$H_{ep} = g\mathop \sum \limits_{i\sigma } n_{i\sigma } \left( {b_{i}^{\dag } + b_{i} } \right) ,$$

where $H_{e}$ describes the Hubbard Hamiltonian, $H_{p}$ is the phonon Hamiltonian and $H_{ep}$ is the Holstein e-p interaction. In Eq. (2), the parameter $t$ is the nearest-neighbour hopping integral, the operator $c_{i\sigma }^{\dag } \left( {c_{i\sigma } } \right)$ creates (annihilates) a spin-$\sigma$ electron at the $i$–th site, $n_{i\sigma } \left( { = c_{i\sigma }^{\dag } c_{i\sigma } } \right)$ being the corresponding electron occupation number and $U$ gives the onsite e-e interaction energy. In Eq. (3), $b_{i}^{\dag } (b_{i} )$ represents an operator that creates (annihilates) an optical phonon at site $i$ with dispersionless frequency ${\omega }_{0},$ ${\lambda }_{ap}$ and $\gamma$ measure respectively the strength and range of the phonon anharmonicity. In Eq. (4), $g$ is the on-site e-p coupling strength which can be written as: $g=\sqrt{\alpha }{\omega }_{0},$ where dimensionless $\alpha$ is referred to as the e-p coupling constant.

## Formulation

### GS energy

In order to solve the Hamiltonian (1) we choose to seek a variational solution. First of all, we apply the modified Lang-Firsov transformation (LFT)^35^ with the generator

5 $$R_{1} = \sqrt \alpha \eta \mathop \sum \limits_{i\sigma } n_{i\sigma } \left( {b_{i}^{\dag } - b_{i} } \right) ,$$

where $\eta$ is the variational parameter that carries the information of the polaronic structure. For strong e-p interaction, $\eta \to 1$, and Eq. (5) generates the usual LFT^36^ and gives a reasonable approximation for the anti-adiabatic region in which the ion motion is much faster than the electron motion. This should work well for the narrow-band materials which are essentially strongly correlated systems. Because of the above transformation, the Hamiltonian $H$ transforms to $H_{1} = e^{{R_{1} }} He^{{ - R_{1} }} .$ To deal with the adiabatic regime where the electron motion is much faster than the ion motion, we perform the Takada-Chatterjee (TC) transformation^10^ with the generator:

6 $$R_{2} = \mathop \sum \limits_{i} h_{i} \left( {b_{i}^{\dag } - b_{i} } \right).$$

where we assume, $h_{i} = h$, as all sites are equivalent. After the second transformation, the transformed Hamiltonian becomes: $H_{2} = e^{{R_{2} }} H_{1} e^{{ - R_{2} }} .$ The above two transformations together can be generated by:

7 $$R_{12} = \mathop \sum \limits_{i\sigma } \left[ {h + \eta \sqrt \alpha \left( {n_{i\sigma } - \frac{h}{\sqrt \alpha }} \right)} \right]\left( {b_{i}^{\dag } - b_{i} } \right).$$

With $\eta = 1,$ the transformation (7) represents the conventional LFT which gives exact results in the anti-adiabatic limit, while for $\eta = 0$, it takes care of the adiabatic limit. Thus both the anti-adiabatic and the adiabatic regions can be studied by considering: $0 < \eta < 1.$ Thus $\eta$ can be called an adiabaticity parameter. It is important to note that Eq. (7) assumes the phonons associated with the electron to be in a coherence state. This is essentially a semi-classical approximation in which it is assumed that the phonons in the polaron cloud are independent of each other satisfying a Poissonian distribution. In other words, the phonons emitted or absorbed by the electrons are completely uncorrelated and in that sense, the present transformation is equivalent to the Hartree approximation.

The presence of Gaussian anharmonicity in the system introduces anharmonicity to infinite order and results in a finite lifetime of the phonons through phonon–phonon interactions. Furthermore, an electron undergoes a recoil motion while emitting a phonon. While undergoing a recoil motion, if the electron emits another phonon, then these two successively emitted virtual phonons will be correlated. Both the anharmonicity and the correlation effects of the phonons can be taken into account by considering squeezed phonons. Squeezing of the phonon vacuum state can be accomplished by the celebrated Bogoliubov transformation with the generator^10^:

8 $$R_{3} = \alpha_{s} \mathop \sum \limits_{i} \left( {b_{i} b_{i} - b_{i}^{\dag } b_{i}^{\dag } } \right),$$

where the squeeze parameter $\alpha_{s}$ is to be obtained variationally. The transformed Hamiltonian is now given by: $H_{3} = e^{{R_{3} }} H_{2} e^{{ - R_{3} }}$ . The variational parameter $\alpha_{s}$ has been considered by all investigators as independent of the electron concentration until 2019, when Malik, Mukhopadhyay and Chatterjee (MMC)^26^ considered the squeezing of the phonon state to be partly dependent on electron density. According to MMC, the correlation between phonons emitted by the electrons may depend on the number of electrons available at a particular lattice site. Thus, to be more realistic, we next apply, a squeezing transformation with the generator:

9 $$R_{4} = \alpha_{d} \mathop \sum \limits_{i} n_{i\sigma } \left( {b_{i} b_{i} - b_{i}^{\dag } b_{i}^{\dag } } \right),$$

where $\alpha_{d}$ is the variational parameter. Correspondingly, the transformed Hamiltonian can be written as: $H_{4} \equiv {\mathcal{H}} = e^{{R_{4} }} H_{3} e^{{ - R_{4} }} .$ It may be pointed out that in Eq. (8), phonon correlation and anharmonicity have been included at a mean-field level while Eq. (9) incorporates the fluctuations. The principal idea of performing a series of canonical transformation on $H$ is to disentangle the electrons and phonons and if the disentanglement is accomplished, then the Hilbert space containing the electronic and phonon variables will become separable. In a simpler language, the purpose of the transformations is to obtain a transformed Hamiltonian in which the electron and phonon variables will be decoupled. However, the Hamiltonian ${\mathcal{H}}$ is still not exactly soluble but it is quite reasonable to assume that after performing the four canonical transformations with generators (5), (6), (8) and (9), the electrons and phonons are weakly entangled in the Hamiltonian ${\mathcal{H}}$ and therefore the eigenstate of ${\mathcal{H}}$ can be approximately written as the product of an electronic state $|\psi_{el}\rangle$ (hitherto unknown) and a phonon state ${|{\Phi }_{ph}}\rangle$ which has to be judiciously chosen. Thus the total wave function corresponding to the full Hamiltonian $H$ can be written as:

10 $$\left| { \Psi_{total}\rangle = } \right|\psi_{ph}\rangle \otimes |\psi_{el}\rangle = e^{{ - R_{1} }} e^{{ - R_{2} }} e^{{ - R_{3} }} e^{{ - R_{4} }} \left| {\Phi_{ph} }\rangle \right|\psi_{el}\rangle$$

As we have already mentioned, both adiabatic and anti-adiabatic effects have been incorporated in Eq. (10). In order to project out the effective electronic Hamiltonian, we have to eliminate the phonons from the problem. To accomplish that we need to take the expectation value of the Hamiltonian ${\mathcal{H}}$ in a suitable phonon state $|{\Phi }_{ph}\rangle$. Usually a zero-phonon state is chosen for the averaging phonon state $|{\Phi }_{ph}\rangle$. To make the calculation most accurate, we choose the averaging phonon state as:

11 $$|{\Phi_{ph}\rangle = \mathop \sum \limits_{n = 0}^{M} r_{n} } |\varphi_{n} \left( x \right)\rangle ,$$

where $\varphi_{n} \left( x \right)$ is the $n -$ th excited-state oscillator eigen function in 1D and the coefficients $r_{n}$’s are to be obtained variationally. The idea is to start the calculation with $M = 0$ and then keep on increasing the value of $M$ till the energy converges. It may be noted that the canonical transformations performed on Hamiltonian $H$ by the generators given by Eqs. (5), (6), (8) and (9), followed by the averaging of the transformed Hamiltonian ${\mathcal{H}}$ with respect to the phonon state (11) is same as taking the expectation value of the Hamiltonian $H$ with respect to the state

12 $$| {\psi_{ph}\rangle = e^{{ - R_{1} }} e^{{ - R_{2} }} e^{{ - R_{3} }} e^{{ - R_{4} }} } |\Phi_{ph}\rangle$$

13 $$= e^{{ - \mathop \sum \limits_{i\sigma } \left[ {h + \eta \sqrt \alpha \left( {n_{i\sigma } - \frac{h}{\sqrt \alpha }} \right)} \right]\left( {b_{i}^{\dag } - b_{i} } \right)}} e^{{ - \alpha_{s} \mathop \sum \limits_{i} \left( {b_{i} b_{i} - b_{i}^{\dag } b_{i}^{\dag } } \right)}} e^{{ - \alpha_{d} \mathop \sum \limits_{i} n_{i\sigma } \left( {b_{i} b_{i} - b_{i}^{\dag } b_{i}^{\dag } } \right)}} \mathop \sum \limits_{n = 0}^{M} r_{n} |\varphi_{n} \left( x \right)\rangle$$

which is the variational state for the phonon sub-system. We choose units in which $\hbar = \omega_{0} = 1,$ $\omega_{0}$ being the dispersionless phonon frequency. The effective electronic Hamiltonian can be defined as: $H_{{eff}} = \langle\psi _{{ph}} |H|\psi _{{ph}}\rangle = \langle\Phi _{{ph}} |{\mathcal{H}}|\Phi _{{ph}}\rangle.$ The expressions for $H_{1} ,$ $H_{2} ,$ $H_{3}$ and $H_{4}$ are rather complicated and long. We therefore do not present their expressions here. $H_{eff}$ assumes the following expression:

14 $$\begin{aligned} H_{eff} & = \langle\psi_{ph} |H|\psi_{ph}\rangle = \langle\Phi_{ph}|{\mathcal{H}}|\Phi_{ph}\rangle =\langle \Phi_{ph} |e^{{R_{4} }} e^{{R_{3} }} e^{{R_{2} }} e^{{R_{1} }} He^{{ - R_{1} }} e^{{ - R_{2} }} e^{{ - R_{3} }} e^{{ - R_{4} }} |\Phi_{ph} \\ & \rangle = \varepsilon_{eff} \mathop \sum \limits_{i\sigma } n_{i\sigma } - t_{eff} \mathop \sum \limits_{{\left\langle {ij} \right\rangle \sigma }} c_{i\sigma }^{\dag } c_{j\sigma } + U_{eff} \mathop \sum \limits_{i} n_{i \uparrow } n_{i \downarrow } + N \lambda_{ap} E_{1} + N\left( {h^{2} - \frac{1}{2}} \right) \\ & \quad + \frac{N}{4}\left[ {S_{2} \left( {1 + 4\alpha_{d} + 12\alpha_{d}^{2} } \right)e^{{4\alpha_{s} }} - S_{3} \left( {1 - 4\alpha_{d} + 12\alpha_{d}^{2} } \right)e^{{ - 4\alpha_{s} }} - 4he^{{2\alpha_{s} }} S_{1} \left( {1 + 2\alpha_{d} + 3\alpha_{d}^{2} } \right)} \right], \\ \end{aligned}$$

where

15 $$\varepsilon_{eff} = - \alpha \eta \left( {2 - \eta } \right) - \sqrt \alpha \left( {1 - \eta } \right)\left[ {e^{{2\alpha_{s} }} S_{1} \left( {1 + 2\alpha_{d} + 3\alpha_{d}^{2} } \right) - 2h} \right] + \lambda_{ap} \left( {E_{2} - E_{1} } \right),$$

16 $$t_{eff} = t M_{1}^{2} M_{2}^{2} ,$$

17 $$U_{eff} = U - 2\alpha \eta \left( {2 - \eta } \right) + \lambda_{ap} \left( {E_{1} - 2E_{2} + E_{3} } \right),$$

with

18 $$\begin{gathered} S_{i} = \mathop \sum \limits_{k,l = 0}^{M} r_{kl} \mathop \smallint \limits_{ - \infty }^{\infty } e^{{ - y^{2} }} \xi_{i} \left( y \right)H_{k} \left( y \right)H_{l} \left( y \right)dy , E_{i} = e^{{ - \gamma \nu_{i} }} F \mathop \sum \limits_{k,l = 0}^{M} r_{kl} \mathop \smallint \limits_{ - \infty }^{\infty } e^{{\left( {\sqrt 2 \zeta_{i} - y} \right)y}} H_{k} \left( y \right) H_{l} \left( y \right)dy ,\hfill \\ M_{1} = \mathop \sum \limits_{k,l = 0}^{M} r_{kl} e^{{-\frac{{a^{2} }}{4}}} \mathop \smallint \limits_{ - \infty }^{\infty } e^{{ -{y^{2} }}} H_{k} \left( {y+\frac{a}{2}} \right) H_{l} \left( {y-\frac{a}{2}} \right)dy, \hfill \\ M_{2} = \mathop \sum \limits_{k,l = 0}^{M} r_{kl} e^{{\alpha_{d} }} \mathop \smallint \limits_{ - \infty }^{\infty } e^{{ - \frac{{y^{2} }}{2}\left( {1 + \beta^{2} } \right)}} H_{k} \left( y \right) H_{l} \left( {y\beta } \right)dy, F = \mathop \sum \limits_{k,l = 0}^{M} r_{kl} \mathop \smallint \limits_{ - \infty }^{\infty } e^{{\left( {2B - 1} \right)y^{2} }} H_{k} \left( y \right)H_{l} \left( y \right)dy, \hfill \\ \end{gathered}$$

where $H_{k} \left( y \right)$ and $H_{l} \left( y \right)$ are the Hermite polynomials of degree ‘$k$’ and ‘$l$’, respectively, and

19 $$\begin{gathered} r_{kl} = r_{k} r_{l} \sqrt {1/2^{k + l} k!l!\pi } , \beta = 1 + 2\alpha_{d} , a = \sqrt {2\alpha } \eta e^{{ - 2\alpha_{s} }} \left( {1 - 2\alpha_{d} + 3\alpha_{d}^{2} } \right), \hfill \\ \xi_{1} = \sqrt 2 y,\xi_{2} = 2y^{2} ,\xi_{3} = 2\left( {y^{2} - 2l - 1} \right), \zeta_{i} = 2\gamma \nu_{i} e^{{2\alpha_{s} }} \left( {1 + 2\alpha_{d} + 3\alpha_{d}^{2} } \right) \hfill \\ \nu_{1} = 2h, \nu_{2} = 2\left( {h + \sqrt \alpha \eta } \right), \nu_{3} = 2\left( {h + 2\sqrt \alpha \eta } \right), B = \gamma e^{{4\alpha_{s} }} \left( {1 + 4\alpha_{d} + 12\alpha_{d}^{2} } \right). \hfill \\ \end{gathered}$$

The GS energy of the system described by the Hamiltonian $H_{eff}$ can be obtained exactly at half-filling with the help of the Bethe ansatz method first implemented by LW^11^. The LW solution has however been obtained for $U_{eff} > 0.$ We modify the solution to include the results for $U_{eff} \le 0$^10,38^. The GS energy per electron $\left( \varepsilon \right)$ is finally obtained as

20 $$\begin{aligned} \varepsilon & = - J + \frac{1}{4}\left( {U_{eff} - \left| {U_{eff} } \right|} \right) + \frac{{e^{{4\alpha_{s} }} }}{4} S_{2} \left( {1 + 4\alpha_{d} + 12\alpha_{d}^{2} } \right) - \frac{{e^{{ - 4\alpha_{s} }} }}{4} S_{3} \left( {1 - 4\alpha_{d} + 12\alpha_{d}^{2} } \right) \\ & \quad + \left( {h^{2} + \lambda_{ap} E_{1} - \frac{1}{2}} \right) - he^{{2\alpha_{s} }} S_{1} \left( {1 + 2\alpha_{d} + 3\alpha_{d}^{2} } \right) - \mathop \smallint \limits_{0}^{\infty } \frac{{4 t_{eff} J_{0} \left( y \right)J_{1} \left( y \right)dy}}{{y\left[ {1 + \exp \left( {\frac{{y\left| {U_{eff} } \right|}}{{2t_{eff} }}} \right)} \right]}} , \\ \end{aligned}$$

where

21 $$J = \alpha \eta \left( {2 - \eta } \right) + \sqrt \alpha \left( {1 - \eta } \right)\left[ {e^{{2\alpha_{s} }} S_{1} \left( {1 + 2\alpha_{d} + 3\alpha_{d}^{2} } \right) - 2h} \right] - \lambda_{ap} \left( {E_{2} - E_{1} } \right).$$

$\varepsilon$ is finally minimized numerically with respect to the variational parameters to obtain the GS energy.

### Average lattice displacement ($\langle {\varvec{x}}_{{\varvec{i}}} \rangle$), Entanglement Entropy (EE) and Local Spin Angular Momentum (${{\varvec{L}}}_{0}$)

In this sub-section we attempt to calculate a few interesting quantities such as the Average lattice displacement, Entanglement entropy and Local spin angular momentum ${L}_{0}$ for the system under consideration. These quantities give in general the physical properties of the system. The Entanglement entropy and Local spin angular momentum, in particular, provide information about the different phases that the system may possess. The numerical results for these quantities with respect to different physical parameters of the system will be presented in “Numerical results and discussion”.

The average lattice displacement is calculated with respect to the variational state $|{\uppsi }_{\mathrm{ph}}\rangle$. The result is obtained as

22 $$\langle x_{i} \rangle = \frac{1}{\sqrt 2 }\left[ {S_{1} e^{{2\alpha_{s} }} \left( {1 + 2\alpha_{d} + 3\alpha_{d}^{2} } \right) - 2h - 2\sqrt \alpha \eta } \right] .$$

In order to study the role of quantum correlation in phase transition, we calculate the von Neumann Entanglement entropy for the 1D HH Hamiltonian. Considering a set of four available states $|0\rangle, \left| { \uparrow \rangle, } \right| {\downarrow \rangle}$ and $|\uparrow \downarrow\rangle$, the single-site entanglement entropy is calculated as:

23 $$E_{\vartheta } = - Tr\left( {\rho_{r} \log_{2} \rho_{r} } \right),$$

where $\rho_{r}$ is called the reduced density operator and is given by

24 $$\rho_{r} = d_{e} \left| {0\rangle\langle0} \right| + d_{ \uparrow } \left| { \uparrow \rangle \langle \uparrow } \right| + d_{ \downarrow } \left| { \downarrow \rangle \langle \downarrow } \right| + d_{ \uparrow \downarrow } \left| { \uparrow \downarrow \rangle \langle\uparrow \downarrow } \right| ,$$

where

25 $$d_{ \uparrow \downarrow } = \langle n_{i \uparrow } n_{i \downarrow }\rangle = d ; d_{ \uparrow } = d_{ \downarrow } = \frac{n}{2} - d_{ \uparrow \downarrow } ; d_{e} = 1 - d_{ \uparrow } - d_{ \downarrow } - d_{ \uparrow \downarrow } .$$

Using the Hellmann–Feynman theorem, we get

26 $$\frac{\partial \varepsilon }{{\partial U}} =\langle n_{i \uparrow } n_{i \downarrow }\rangle = d_{ \uparrow \downarrow } .$$

Thus all the occupation numbers can be calculated and the corresponding von Neumann entanglement entropy $\left( {E_{\vartheta } } \right)$ is evaluated.

The mean-square spin angular momentum per site ($L_{0}$) can be defined as:

27 $$L_{0} = \frac{1}{N}\mathop \sum \limits_{i}\langle S_{i}^{2}\rangle ,$$

where $S_{i}$ is the electron spin at site $i$, and $S_{i}^{2} = S_{ix}^{2} + S_{iy}^{2} + S_{iz}^{2}$. Using $S_{i}^{ \pm } = S_{ix} \pm iS_{iy}$, $S_{i}^{z} = \left( {n_{i \uparrow } - n_{i \downarrow } } \right)/2$, $S_{i}^{ + } = c_{i \uparrow }^{\dag } c_{i \downarrow } ,$ $S_{i}^{ - } = c_{i \downarrow }^{\dag } c_{i \uparrow }$, $S_{i}^{ + } \cdot S_{i}^{ - } = - n_{i \uparrow } n_{i \downarrow }$, $n_{i \uparrow }^{2} = n_{i \uparrow }$, $n_{ i \downarrow }^{2} = n_{i \downarrow }$, we obtain, $S_{i}^{2} = 3\left[ {1 - 2\langle n_{i \uparrow } n_{i \downarrow } \rangle } \right]/4$ and consequently

28 $$L_{0} = \frac{3}{4} - \frac{3}{2N}\mathop \sum \limits_{i} \langle n_{i \uparrow } n_{i \downarrow }\rangle = \frac{3}{4} - \frac{3}{2}\frac{d\varepsilon }{{dU}} .$$

where use has been made of Eq. (26). $L_{0}$ gives a measure of the spin magnetic moment and will be loosely referred to as the spin moment. For a completely un-correlated electron gas, we can write: $\langle n_{i \uparrow } n_{i \downarrow } \rangle = \langle n_{i \uparrow } \rangle \langle n_{i \downarrow } \rangle$, and the average spin moment per site ($L_{0}$) becomes equal to 0.375.

## Numerical results and discussion

### Ground State (GS) Energy

**Figure 1:**
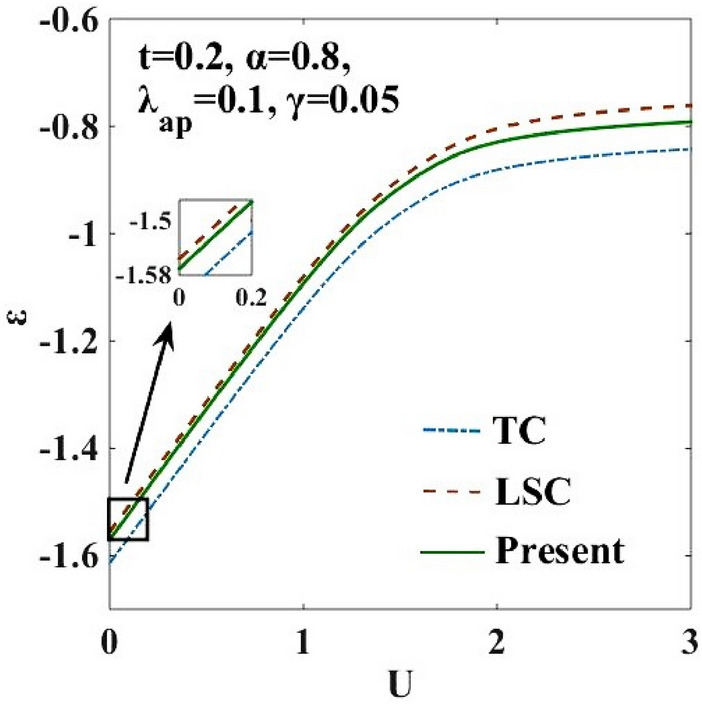
Single-site GS energy $(\varepsilon )$ vs. onsite Coulomb energy (*U*). TC result corresponds to the harmonic case^10^. ‘LSC’ refers to the result of Lavanya et al.^25^ obtained for the anharmonic case with three canonical transformations. “Present” refers to the result for the anharmonic case obtained by including the effect of the new transformation with the generator ${R}_{4}$ in the present work.

The single-site GS energy is determined by varying $\varepsilon$ in the space of the variational parameters. The minimum value of $\varepsilon$ gives the GS energy. The results are shown in Fig. 1. The harmonic TC results $(\lambda =0 \& \gamma =0)$^10^ are also plotted to show the effect of anharmonicity. It is clear that the lattice anharmonicity has a significant effect on the GS energy. We also show the results of the anharmonic case reported recently by Lavanya et al. (LSC)^25^. The LSC results have been obtained by using three canonical transformations. The present work uses an additional transformation namely the transformation given by Eq. (9). For $\lambda =0.1$ and $\gamma =0.05$, we find that the new transformation (9) has only a marginal effect on the GS energy at small $U$. However, at large $U,$ some observable effect is evident. More importantly, as we will show later, it has a more discernible effect on the phase diagram. Here we would like to mention an important point. We know that in a variational calculation, an error in the trial wave function of order $\updelta$ will give an error of the order ${\updelta }^{2}$ in the energy. So, even a reasonable improvement in the wave function may not provide a sizable change in the energy. More importantly, here we are not so much interested in the energy but in the phase diagram. Our interest lies in examining whether with the improvement in the variational wave function, the width of the metallic phase broadens or shortens. If an improved variational calculation which obviously lowers the energy (by whatever amount) narrows the metallic phase, then the claim that an intermediate metallic phase exists at the cross-over region of the CDW-SDW phases may be discounted. Our aim has been to show that with every improved variational calculation (which might improve the ground sate energy only marginally) the intermediate metallic phase widens. This certainly lends credence to the prediction of the existence of the intermediate metallic phase.

### Average lattice displacement

**Figure 2:**
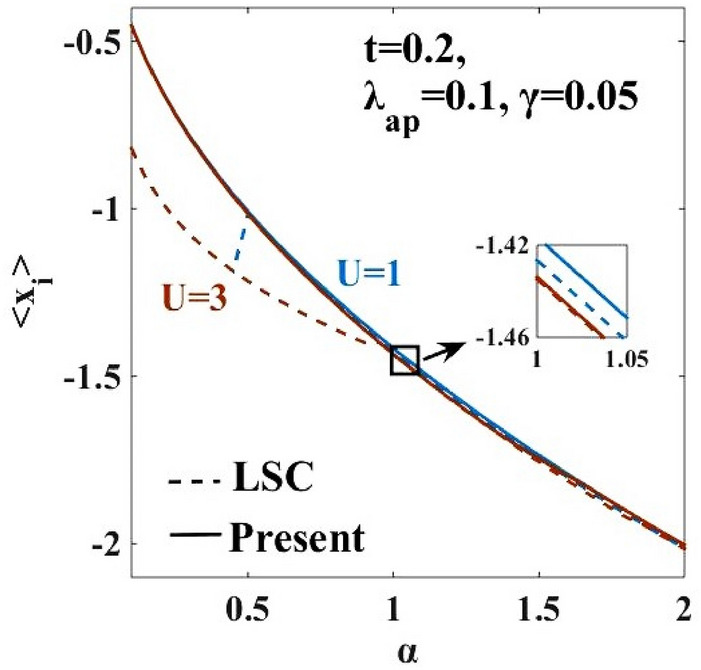
Average lattice displacement (${<x}_{i}>$) vs. e-p interaction coefficient ($\alpha$). ‘LSC’ refers to the result of Lavanya et al.^25^ obtained for the anharmonic case with three canonical transformations. “Present” refers to the result for the anharmonic case obtained by including the effect of the new transformation with the generator ${R}_{4}$ in the present work.

A plot of the average lattice displacement $\left(\langle {x}_{i}\rangle \right)$ versus the e-p coupling constant ($\alpha )$ is shown in Fig. 2. One can see that the magnitude of $\langle {x}_{i}\rangle$ is a decreasing function of $\alpha$. For the harmonic case, Eq. (22) yields $\langle {x}_{i}\rangle =-\sqrt{2\alpha }$ . The negative value of $\langle {x}_{i}\rangle$ signifies that the lattice displacement takes place in the opposite direction of the polaronic motion leading to a mass renormalization of the electron.

### Broadening of the metallic phase

**Figure 3:**
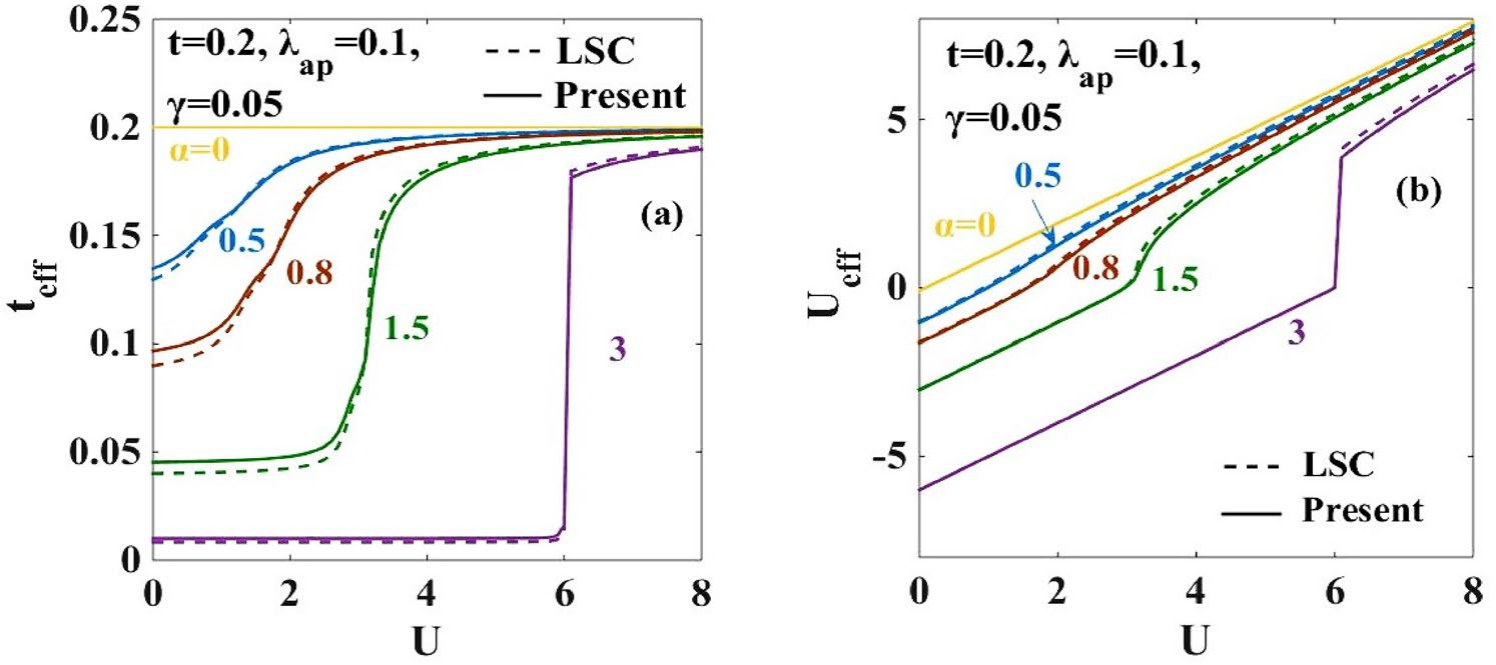
(**a**) Effective hopping parameter ($${t}_{eff})$$ vs on-site Coulomb interaction strength ($$U)$$ ; (**b**) Effective e-e interaction energy $${U}_{eff}$$ vs on-site Coulomb interaction strength $$(U)$$. “LSC” refers to the result for the anharmonic case obtained by Lavanya et al.^25^ using three canonical transformations. “Present” refers to the result obtained for the anharmonic case obtained by including the effect of the new transformation with generator $${R}_{4}$$ in the present work.

The variations of the effective hopping integral $$({t}_{eff})$$ and the effective onsite e-e interaction energy $$({U}_{eff})$$ are respectively studied in Fig. 3a,b with respect to the onsite Coulomb energy ($$U)$$ for the different strengths of the e-p coupling strength $$(\alpha )$$. As expected, for $$\alpha =0$$, $${t}_{eff}$$ becomes equal to the bare Hubbard hopping parameter $$t$$ and $${U}_{eff}$$ becomes equal to the Hubbard $$U.$$ As $$\alpha$$ increases, $${t}_{eff}$$ decreases and with the increase in $$U$$, it gradually increases and saturates to the Hubbard value. At small values of $$U$$, the effective attractive onsite e-e interaction induced by the e-p interaction overcomes the repulsive Coulomb interaction $$U$$ and therefore $${U}_{eff}$$ becomes negative, i.e., attractive. The lattice is then unstable against the Peierls transition in which bound states of singlet bipolarons form on every alternate site leading to an insulating phase called the CDW state. On the contrary, when $$U$$ is larger compared to $$\alpha$$, the repulsive onsite electronic interaction wins, and the polarons cannot hop from one site to the other and consequently the GS of the system is given by the AFM mott-insulator state which is also known as SDW. Figure 3a shows that in the weak e-p interaction regime, the variation of $${t}_{eff}$$ and $${U}_{eff}$$ with $$U$$ is continuous. But for higher values of $$\alpha ,$$ discontinuous jumps occur in the behaviour of $${t}_{eff}$$ and $${U}_{eff}.$$ The discontinuous jump corresponds to a direct CDW-SDW transition.

**Figure 4:**
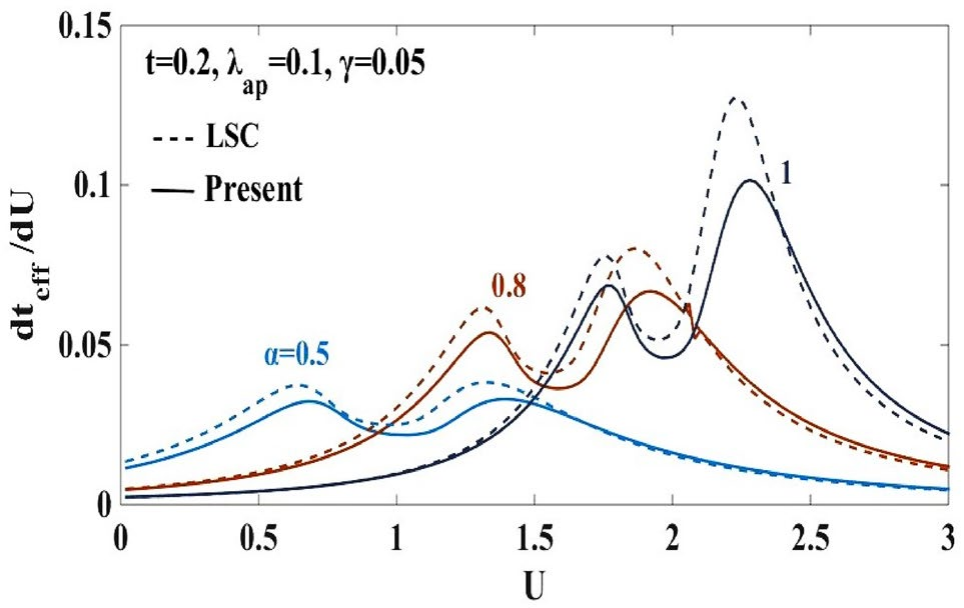
$d{t}_{eff}/dU$ vs. onsite e-e inetraction $U$ for different e-p interaction strengths $\alpha =0.5, 0.8, 1.0$. “LSC” refers to the result for the anharmonic case obtained by Lavanya et al.^25^ using three canonical transformations. “Present” refers to the result obtained for the anharmonic case obtained by including the effect of the new transformation with generator ${R}_{4}$ in the present work.

**Figure 5:**
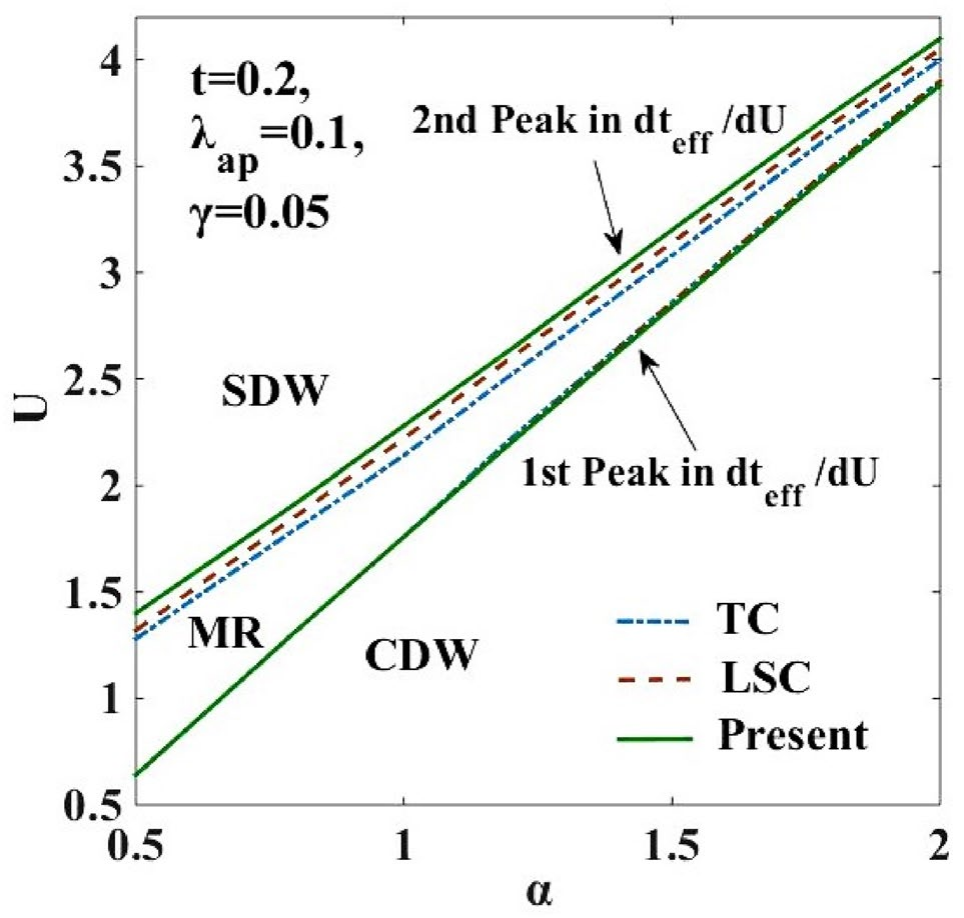
Phase diagram in the ($\alpha -U)$ plane. TC refers to result for the harmonic case obtained by Takada and Chatterjee^10^. “LSC” refers to the result for the anharmonic case obtained by Lavanya et al.^25^ using three canonical transformations. “Present” refers to the result obtained for the anharmonic case obtained by including the effect of the new transformation with generator ${R}_{4}$ in the present work. MR refers to the metallic region.

In order to examine the nature of the transition between the two phases observed in Fig. 3a,b, the quantity, ($$d{t}_{eff}/dU)$$ is plotted in Fig. 4 with respect to $$U$$ for $$\alpha =0.5, 0.8$$ and $$1.0$$. The double-peak structure in $$(d{t}_{eff}/dU)$$ is clearly evident. One can also observe that the peaks grow in height and shift towards larger values of $$U$$ with increasing $$\alpha .$$ Furthermore, the new transformation used in the present calculation broadens the width between the two peaks in Fig. 4. Corresponding to the peak values of the $$(d{t}_{eff}/dU)$$ vs $$U$$ plot, the phase diagram is drawn in the $$(\alpha -U)$$ plane. This is shown in Fig. 5. The intermediate phase satisfies the condition: $$4{t}_{eff}\ge {U}_{eff}$$ , which is the signature of a metallic or a conducting phase. The metallic phase is flanked by the SDW phase on the left and the CDW phase on the right. The figure shows that the intermediate metallic phase appearing at the CDW-SDW cross-over region is now wider compared to that predicted by LSC^25^. It is important to emphasize that it is not important by how much the present modified variational wave function broadens the width of the intermediate region, that the improved variational calculation widens the metallic phase is itself a result of great significance. The reason is simple. If a modified variational wave function predicts a narrower intermediate phase, it will have a disastrous effect on the prediction of the existence of a metallic region (MR) because one may then argue that the metallic phase may as well collapse if a more improved variational calculation is performed. That with every improved variational calculation, the metallic phase widens is indeed an encouraging result.

**Figure 6:**
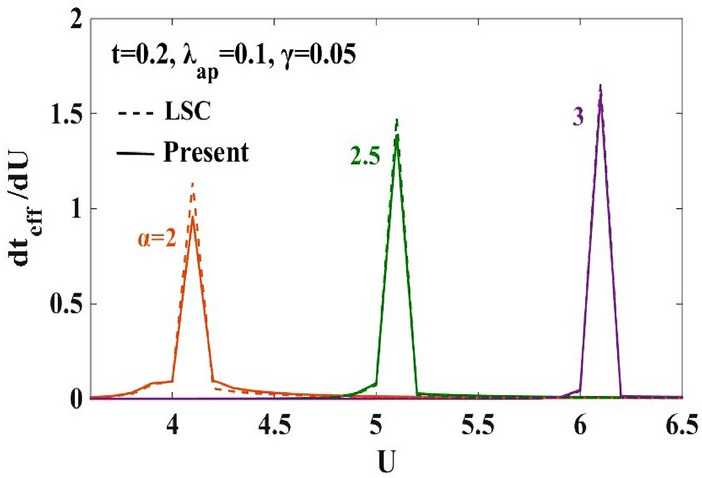
$d{t}_{eff}/dU$ vs. onsite e-e inetraction $U$ for different e-p interaction strengths ( $\alpha =2.0, 2.5, 3.0)$. “LSC” refers to the result for the anharmonic case obtained by Lavanya et al.^25^ using three canonical transformations. “Present” refers to the result obtained for the anharmonic case by including the effect of the new transformation with generator ${R}_{4}$ in the present work.

In Fig. 6, we plot $(d{t}_{eff}/dU)$ with $U$ for larger values of α. We find that the double peak structure almost disappears as $\alpha$ increases and one can observe from Fig. 6 that for $\alpha >2$, only a single peak structure appears.

This indicates the absence of any intermediate phase for large α.

**Figure 7:**
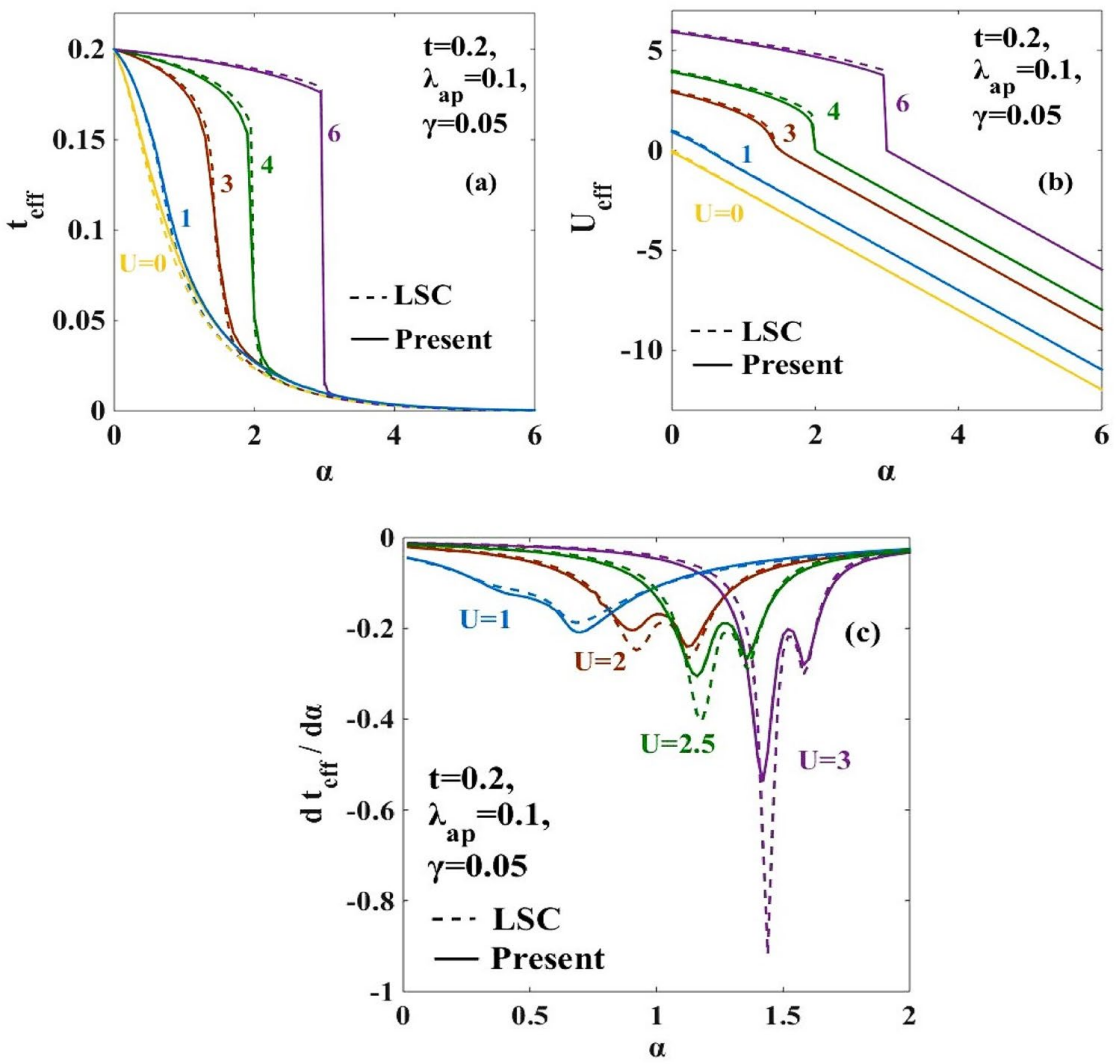
(**a**) Effective hopping parameter (${t}_{eff}$) vs. e-p interaction coefficient $(\alpha )$ for different values of $U$; (**b**) Effective e-e interaction (${U}_{eff}$) vs. e-p interaction coefficient ($\alpha )$ for different values of $U;$ (**c**) $(d{t}_{eff}/d\alpha$) vs. $\alpha$ for different values of $U.$ “LSC” refers to the result for the anharmonic case obtained by Lavanya et al.^25^ using three canonical transformations. “Present” refers to the result obtained for the anharmonic case obtained by including the effect of the new transformation with generator ${R}_{4}$ in the present work.

Figure 7a,b illustrate respectively the behaviour of ${t}_{eff}$ and ${U}_{eff}$ as a function of $\alpha$. As $\alpha$ $\to 0,$ ${t}_{eff}\to t$. Thus at low $\alpha ,$ the system GS is in the SDW phase. As $\alpha$ increases, ${t}_{eff}$ gradually decreases and finally falls off to zero. Figure 7b tells us that corresponding ${U}_{eff}$ becomes maximally negative. This indicates the formation of massive singlet bipolarons giving rise to the CDW phase. Here also we see that for large $U,$ SDW-CDW transition is again direct.

The phase boundaries of the $(\alpha -U)$ phase diagram can also be verified by plotting ($d{t}_{eff}/d\alpha )$ with respect to the e-p interaction coefficient $\alpha$. This is shown in Fig. 7c. The peak-like structure is again obtained in the ($d{t}_{eff}/d\alpha )$ vs $\alpha$ plot for different $U$ values and the peak width for the present result is broader than that of the LSC result. This again proves the intermediate metallic phase is widened.

### Double occupancy $(d)$ and EE ${(E}_{\upsilon })$

**Figure 8:**
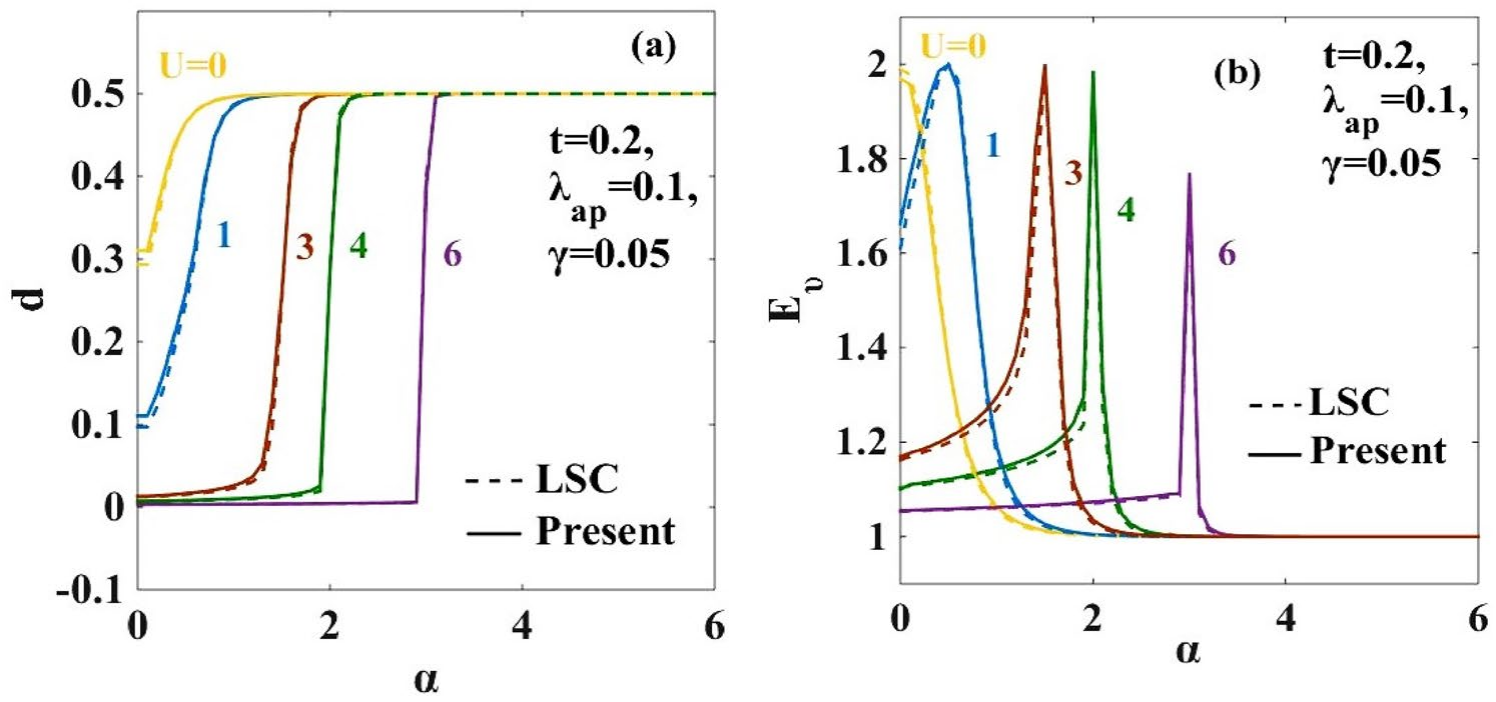
(**a**) Double occupancy parameter $(d)$ vs. e-p interaction coefficient $(\alpha )$ for different values of $U$; (**b**) Entanglement entropy $({E}_{\nu })$ vs. e-p interaction coefficient $(\alpha )$ for different values of $U.$ “LSC” refers to the result for the anharmonic case obtained by Lavanya et al.^10^ using three canonical transformations. “Present” refers to the result obtained for the anharmonic case obtained by including the effect of the new transformation with generator ${R}_{4}$ in the present work.

**Figure 9:**
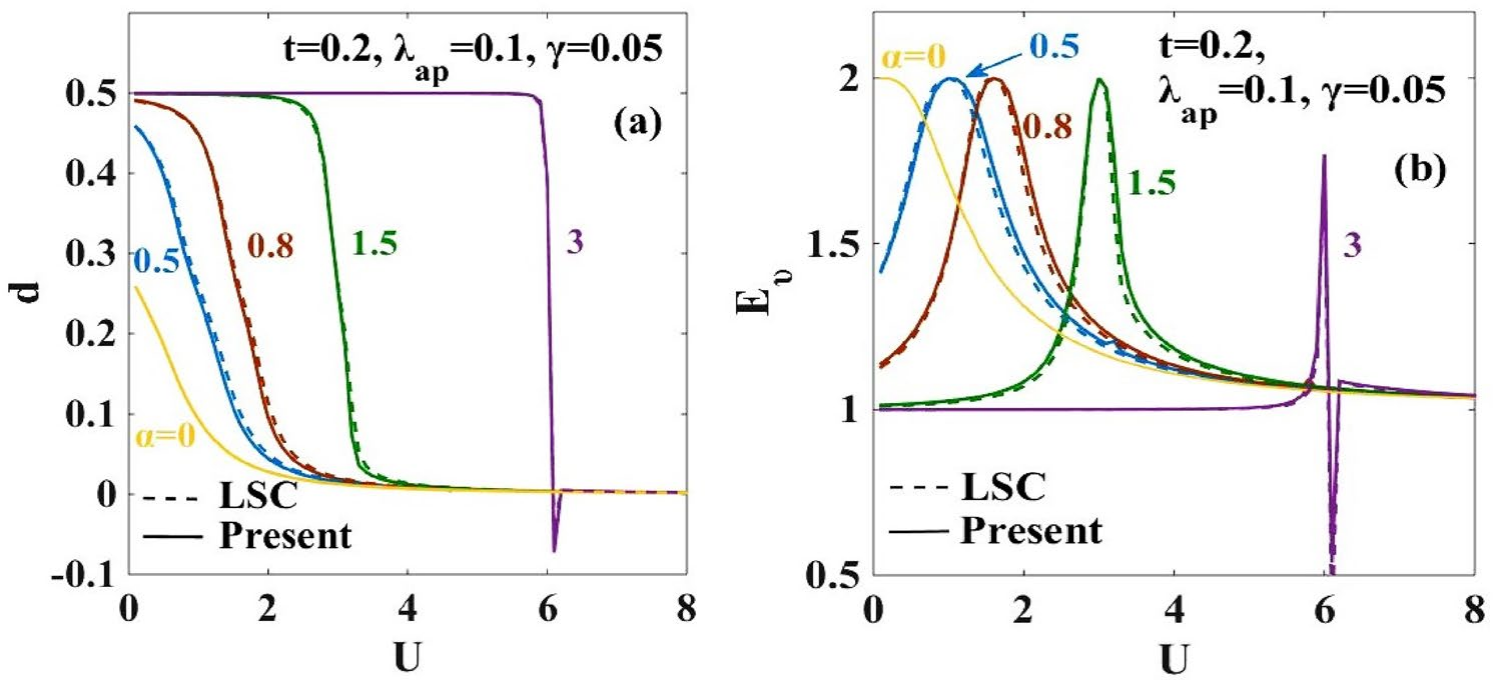
(**a**) Double occupancy parameter $(d)$ vs. onsite Coulomb interaction energy (U) for different values of $\alpha$; (**b**) Entanglement entropy (${E}_{\nu })$ vs. e-e Coulomb interaction energy (U) for different values of $\alpha$. “LSC” refers to the result for the anharmonic case obtained by Lavanya et al.^10^ using three canonical transformations. “Present” refers to the result obtained for the anharmonic case obtained by including the effect of the new transformation with generator ${R}_{4}$ in the present work.

In Fig. 8a,b, we plot respectively the double occupancy $(\mathrm{d})$ and quantum EE $({\mathrm{E}}_{\upnu })$ as a function of $\mathrm{\alpha }.$ The entanglement entropy (EE) gives a measure of the accessible states the system can have. Obviously then, the maximum in EE would correspond to a conducting state. It is observed that for certain combinations of $\mathrm{\alpha }$ and $U$, ${\mathrm{E}}_{\upnu }$ has maxima and for other values ${\mathrm{E}}_{\upnu }$ becomes very small. Small values of EE correspond to insulating states. When the e-p interaction becomes strong compared to the e-e interaction, the electrons form pairs and the double occupancy parameter $d$ reaches the maximum value of 0.5 driving the system to the CDW state. For $\mathrm{d}<0.5$, the formation of a polaronic SDW state takes place. Similar behaviour is observed in Fig. 9a,b when we plot the double occupancy $(\mathrm{d})$ and quantum EE $({\mathrm{E}}_{\upnu })$ with respect to $U$ for different values of e-p interaction ($\alpha$).

**Figure 10:**
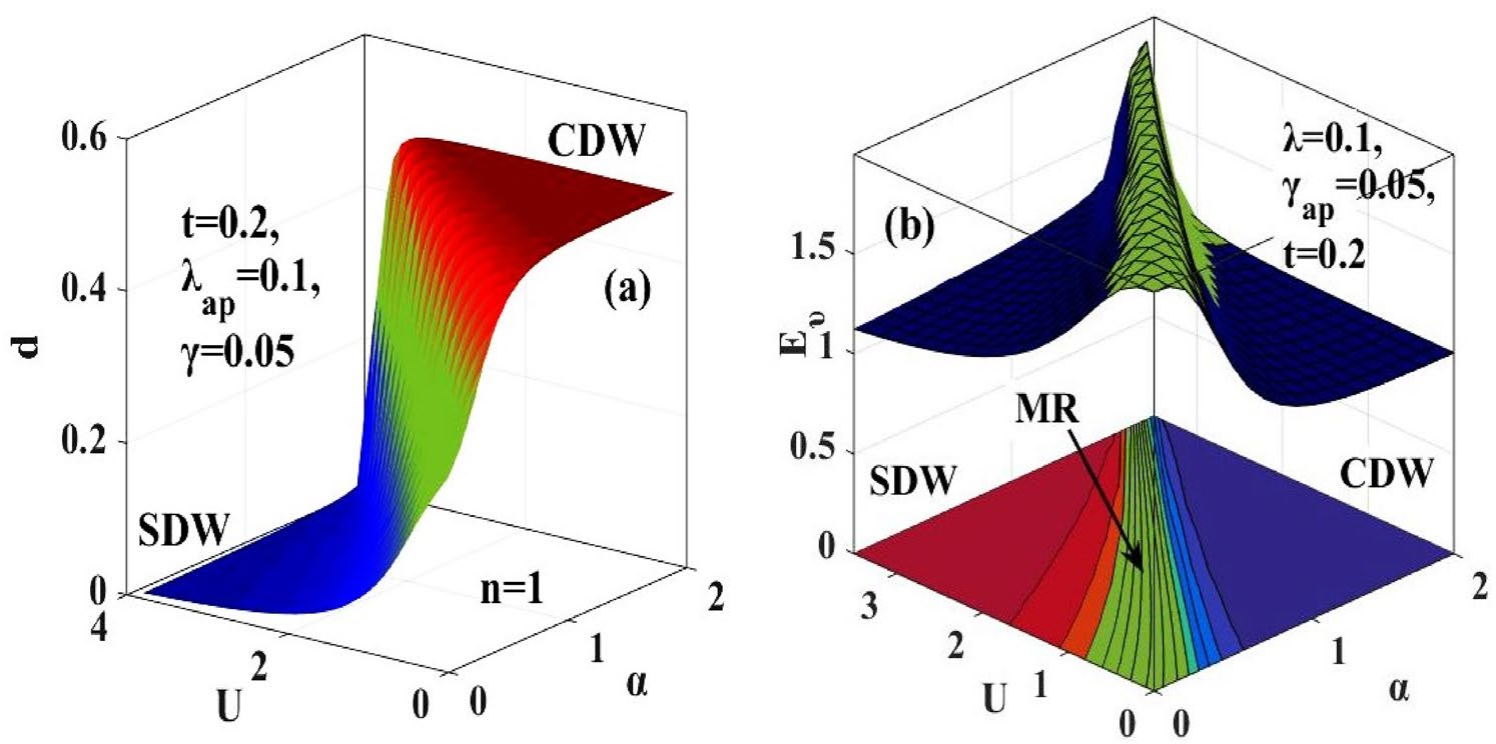
(**a**) 3D plot of double occupancy parameter $(d)$ vs. e-p interaction coefficient $(\alpha )$ and e-e interaction coefficient $(U)$; (**b**) 3D plot of Entanglement Entropy $({E}_{\upsilon })$ vs. $\alpha$ and $U$. MR refers to the metalic phase.

In order to unravel the effect of e-p and e-e interaction simultaneously, we plot 3D graphs in Fig. 10. Figure 10a shows that between the SDW and CDW phases, there lies a region where the value of $d$ neither corresponds to the SDW region with $d=0$ nor to the CDW region with $d=0.5.$ Therefore, the effect of e-p and e-e interactions has been found simultaneously on $d$ and ${\mathrm{E}}_{\upnu }.$ This intermediate cross-over region corresponds to the metallic phase. In Fig. 10b, the peak of EE (${\mathrm{E}}_{\upnu })$ lies over the metallic region (MR) in the $(\mathrm{\alpha }-\mathrm{U})$ plane. Therefore, the peak denotes the metallic phase.

### Mott–Hubbard (MH) criterion in 3D plots

**Figure 11:**
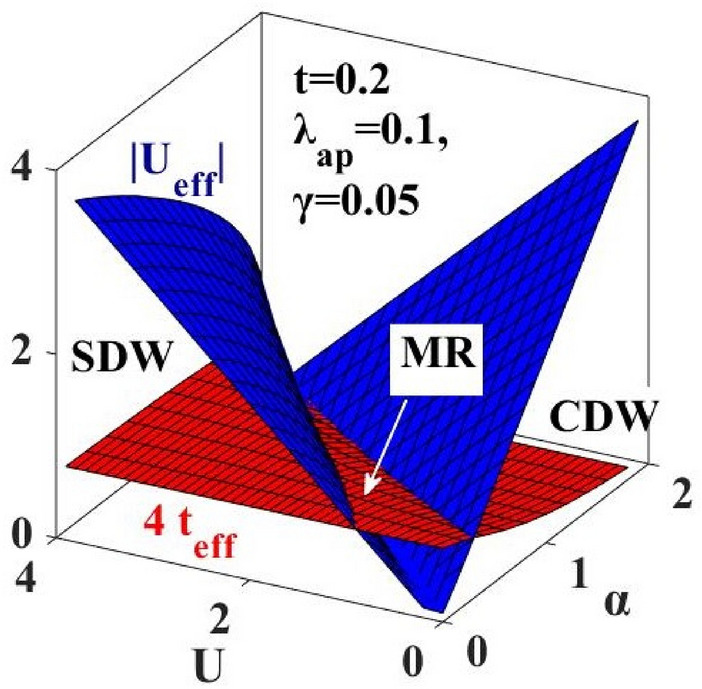
Study of the Mott–Hubbard (MH) criteria for the metallic region : 3D plots of $4{t}_{eff}$ and $|{U}_{eff}|$ with respect to $\alpha$ and $U$.

**Figure 12:**
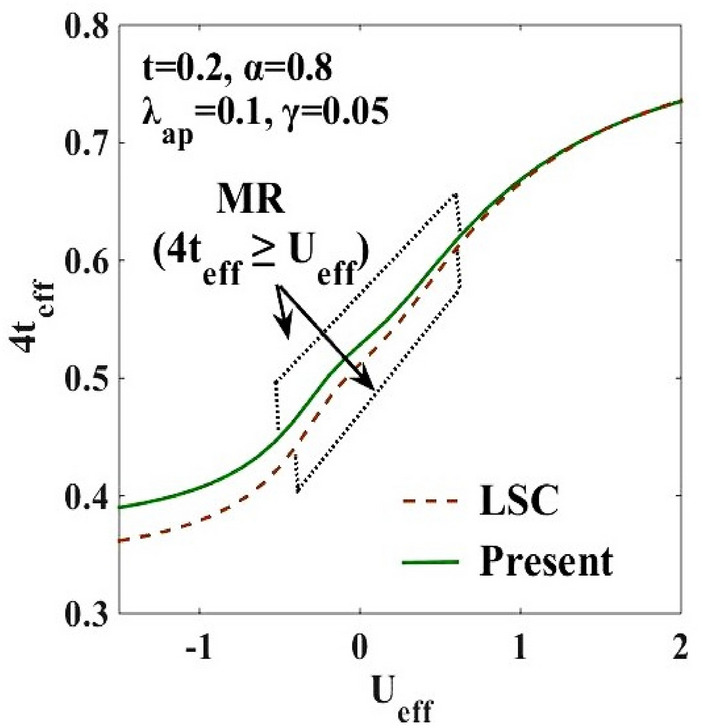
Broadening of Mott–Hubbard (MH) region (MR): $4{t}_{eff}$ vs ${U}_{eff}$.

We have already emphasized that for a metallic state the bandwidth follows the criterion: $2z{t}_{eff}\ge {U}_{eff}$. In Fig. 11, we present a 3D representation of $|{U}_{eff}|$ and $4{t}_{eff}$ with respect to $U$ and $\alpha$. The figure displays a region of $(\alpha , U)$ where the condition: $4{t}_{eff}\ge {U}_{eff}$ is satisfied. This is the metallic phase. There are two other regions in the $\left(\alpha -U\right)$ plane where this condition is not satisfied and those are insulating phases. Among them the phase where ${U}_{eff}>0$ corresponds to the SDW phase and the one where ${U}_{eff}<0$ corresponds to the CDW state. In order to look into the metallic region by the Mott–Hubbard criterion more directly, $4{t}_{eff}$ is plotted in Fig. 12, with respect to ${U}_{eff}$ and the region that satisfies the Mott–Hubbard condition is indicated by the dotted line. It is observed that the region satisfying the Mott–Hubbard criterion is more extended in the present work than the LSC result, which again confirms the broadening of the intermediate metallic region.

### Local Spin Moment

**Figure 13:**
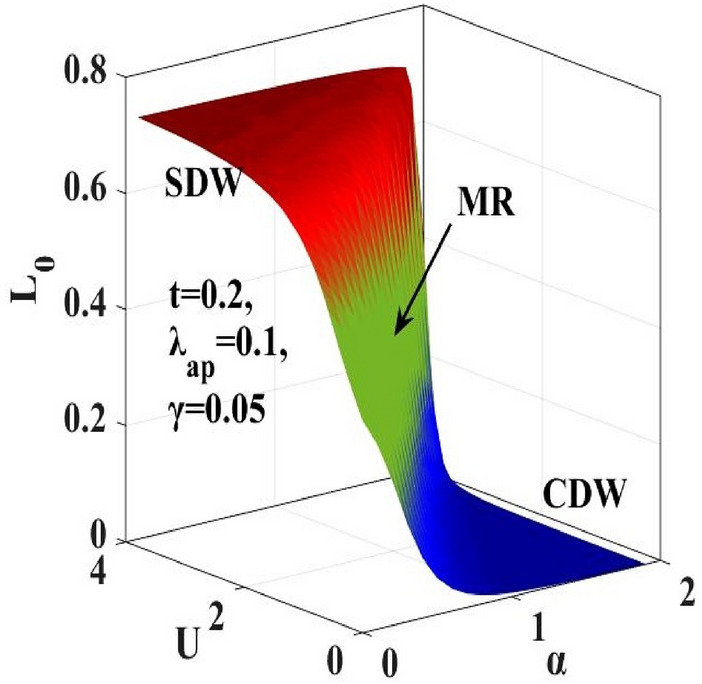
Local spin moment $({L}_{0})$ vs. e-p interaction coefficient $(\alpha )$ and e-e interaction coefficient $(U).$

**Figure 14:**
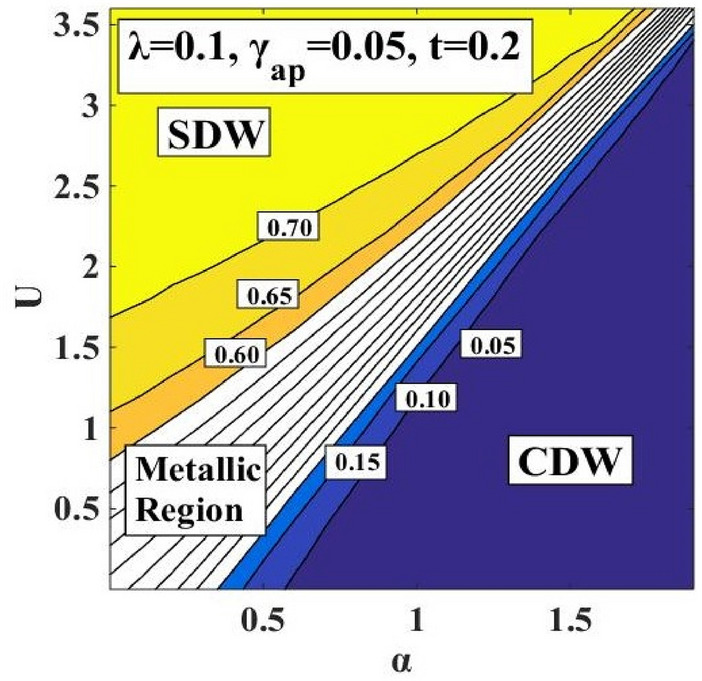
Contour plot of Local spin moment $({L}_{0})$ in the $\left(\alpha -U\right)-$ plane.

The local spin moment $({L}_{0})$ is calculated using Eq. (28) and plotted in Fig. 13 with respect to $\alpha$ and $U$ and in Fig. 14, the contour plots for constant ${L}_{0}$ are drawn in the $\left(\alpha -U\right)$ plane. Both the figures indicate the presence of an intermediate metallic phase which is consistent with the phase diagram. The contour plot in LSC’s work predicts a metallic region (MR) to lie between ${L}_{0}=0.25$ and ${L}_{0}=$ 0.50 while the present calculation shows an extended metallic region (MR) that lies between ${L}_{0}=0.15$ and ${L}_{0}=$ 0.60. One can make the same observation from the local spin moment calculation that re-establishes the broadening of the intermediate metallic region between the CDW and SDW regions.

## Conclusion

The nature of SDW-CDW transition has been studied in a 1D half-filled Holstein-Hubbard model with Gaussian phonon anharmonicity by improving the variational calculation of Lavanya et al.^10^. Using a number of unitary transformations performed in succession followed by a generalized many-phonon averaging an effective electronic Hamiltonian is obtained. The phonon-subsystem has been treated in a semi-exact way. The effective electronic Hamiltonian has been solved exactly using the Bethe ansatz technique to obtain the ground state energy. The hopping integral and the Coulomb correlation are renormalized by the e-p interaction and phonon anharmonicity. Using the Mott–Hubbard criterion we have shown that the present modified approach broadens the width of the intermediate metallic phase reported by Lavanya et al.^10^. The same conclusion has been drawn from the calculation of the local spin moment. Finally, a study of the quantum entanglement entropy and the double occupancy parameter reconfirms the existence of a wider intermediate metallic region at the SDW-CDW transition region.
